# Skin Microcolumns as a Source of Paracrine Signaling Factors

**DOI:** 10.1089/wound.2019.1045

**Published:** 2020-02-07

**Authors:** Joshua Tam, Martin Purschke, Christiane Fuchs, Ying Wang, R. Rox Anderson

**Affiliations:** ^1^ Wellman Center for Photomedicine, Massachusetts General Hospital, Boston, Massachusetts.; ^2^ Department of Dermatology, Harvard Medical School, Boston, Massachusetts.

**Keywords:** cytokines, autologous, proliferation, migration, full-thickness, skin microcolumns

## Abstract

**Objective:** We recently developed the approach of using “microcolumns” of autologous full-thickness skin tissue for wound repair. The small size of these micro skin tissue columns (MSTCs, ∼0.5 mm in diameter) allows donor sites to heal quickly without scarring. Treatment with MSTCs significantly accelerate wound healing, and suppled various skin cell types and skin structures to replenish the wound volume. This technology is now starting clinical use. In this study, we investigate whether MSTCs may also influence wound healing by releasing soluble signaling factors.

**Approach:** Freshly harvested MSTCs were incubated in culture medium for 24 h. The conditioned medium was collected and tested for its effects on migration and proliferation of human dermal fibroblasts, and its ability to induce tube formation by human umbilical vein endothelial cells (HUVECs). Proteins released into the conditioned medium were characterized by multiplex enzyme-linked immunosorbent assay (ELISA), and compared with medium conditioned by an equivalent mass of intact full-thickness skin.

**Results:** MSTC-conditioned medium increased fibroblast migration and proliferation, as well as HUVEC tube formation. MSTCs released many soluble factors known to play prominent roles in wound healing. A subset of proteins showed significantly different release profiles compared with intact full-thickness skin.

**Innovation:** The technology for harvesting and using MSTCs to augment wound healing was recently developed as an alternative to conventional autologous skin grafting. This study shows that MSTCs could also function as “cytokine factories.”

**Conclusion:** In addition to supplying autologous cells to repopulate the wound volume, MSTCs can also function as a source of growth factors and cytokines to further enhance wound healing.

**Figure:**
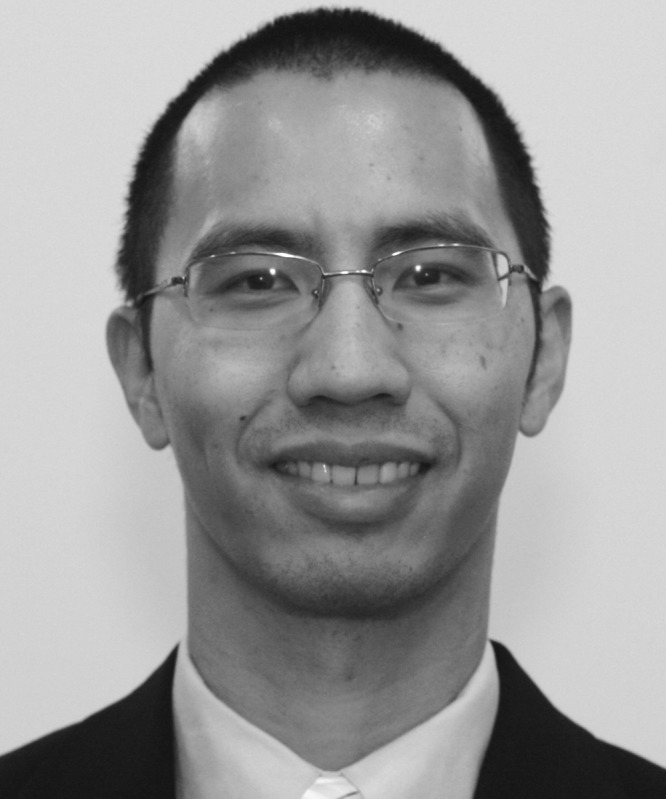
**Joshua Tam, PhD**

## Introduction

Skin wounds and scars constitute a substantial burden to the health care system, with millions of patients affected and wound care-related expenses estimated to cost billions of dollars annually in the United States alone.^1^ Autologous skin grafting, the current gold standard for wound repair, leads to substantial donor-site morbidities,^2,3^ whereas engineered skin substitutes are very costly and as yet unable to fully restore normal skin structure and functions.^4^ We recently developed the new approach of harvesting small “microcolumns” of full-thickness autologous skin tissue for wound repair. The small diameter (about 500 μm) of these micro skin tissue columns (MSTCs) allows the donor site to heal quickly and regenerate without scarring,^5^ which eliminates the principle drawback of autologous skin grafting. In animal studies, skin columns applied directly into skin wounds resulted in accelerated wound healing,^5,6^ stable engraftment of multiple skin cell types, as well as restoration and functionality of adnexal structures^7^—which was hitherto only achieved by using full-thickness skin grafting. The ability of the MSTC donor sites to heal rapidly with minimal donor-site morbidity was confirmed in a recent clinical study.^8^

The wound healing process is heavily influenced by complex signaling networks involving paracrine and endocrine pathways.^9^ It has long been observed that skin grafts can exert a potent stimulatory effect on overall wound healing, even in wound areas that are not in direct contact with the graft, due to the ability of skin grafts to release various soluble factors to promote a healing response from the surrounding tissue. This has led to the suggestion that skin grafts may function in part (in some cases even primarily) through pharmacological mechanisms.^10^ Similarly, the beneficial effects of cell-based therapies, such as stem cells and cultured tissue products, are often due to their ability to serve as “cytokine factories”, rather than any long-term integration of the exogenous cells into the host.^10,11^ In addition to supplying autologous cells to the wound, MSTCs could contribute to wound healing by producing and/or releasing various signaling molecules, but this aspect has not yet been investigated. Furthermore, it has been reported that mincing of skin grafts alters their cytokine production profile, but whether/how this profile may differ between MSTCs and conventional intact skin grafts is unknown. Therefore, we conducted the present study to determine the release profile of soluble factors by MSTCs, and investigate their ability to impact *in vitro* cell function through paracrine signaling.

## Clinical Problem Addressed

MSTC harvesting is a practical method that enables obtaining autologous full-thickness skin with minimal donor-site morbidity, and could have broad clinical applicability for repairing both acute and chronic skin wounds.

## Materials and Methods

### Sample collection

The use of deidentified human skin tissue obtained from abdominoplasties was determined by the Massachusetts General Hospital's Institutional Review Board (IRB) to be exempt from IRB review. MSTCs were harvested from fresh postabdominoplasty skin tissue, using custom-made, dual-tip harvesting needles, which allowed the collection of full-thickness skin columns containing epidermis, full dermis, and some subcutaneous fat, as previously described.^5,7,12^ The needles were made from commercially obtained 21G needle stock, corresponding to an inner diameter of ∼500 μm. Two hundred milligrams of MSTCs was suspended in 10 mL of Dulbecco's modified Eagle's medium (DMEM; 21063-029; Thermo Fisher Scientific, Waltham, MA), and incubated at 37°C with 5% CO_2_ for 24 h. After the incubation period, the conditioned medium was collected, filtered through a 0.2 μm filter, and stored at −80°C until use. This process was repeated for four independent skin samples.

### Migration assay

Human foreskin fibroblasts (HFF1, purchased from ATCC) were cultured and maintained in DMEM supplemented with 10% fetal bovine serum (Life Technologies, Carlsbad, CA) and 1% Pen/Strep solution (10,000 U/mL penicillin, 10,000 μg/mL streptomycin; Life Technologies), and serum starved overnight before the migration assay. Cell migration in response to MSTC-conditioned medium was tested using the Boyden chamber assay (CytoSelect cell migration assay; Cell Biolabs, Inc., San Diego, CA), following the manufacturer's protocol. Briefly, MSTC-conditioned medium was placed in the lower wells of a 24-well plate. Untreated base DMEM was used for control groups. A transwell insert with 8 μm-diameter pores was placed into each well. Around 1.5 × 10^5^ serum-starved HFF1 cells in unsupplemented DMEM were placed into the inside of each insert. After incubating for 24 h under standard cell culture conditions (37°C, 5% CO_2_), media inside the transwell inserts were removed by aspiration. Cells on the bottom of the inserts were detached by treating with cell detachment solution followed by mechanical agitation. The detached cells were incubated with lysis buffer and CyQuant^®^ GR dye solution. The resulting fluorescence, corresponding to the amount of migrated cells, was detected and quantified using a fluorescence plate reader (SpectroMax M5, 480 nm excitation/520 nm emission).

### Proliferation assay

HFF1 cells were also used to evaluate cell proliferation in response to MSTC-conditioned medium using the PrestoBlue Cell Viability Reagent (Thermo Fisher Scientific), following the manufacturer's protocol. Briefly, HFF1 cells were serum starved overnight, plated into a 96-well plate at 6,000 cells/well and incubated with either MSTC-conditioned medium, or unsupplemented DMEM as control for 24 h under standard cell culture conditions. Afterward, PrestoBlue was added to a final 1:10 dilution and incubated for 10 min. The resultant fluorescence at 550 nm excitation/600 nm emission was measured with the SpectroMax spectrophotometer.

### Endothelial cell tube formation assay

Human umbilical vein endothelial cells (HUVECs, purchased from ATCC) were cultured and maintained in Medium 200PRF supplemented with low serum growth supplement (Thermo Fisher Scientific). For the tube formation assay, HUVECs were labeled with Calcein AM dye (Thermo Fisher Scientific), and mixed with Geltrex matrix (Thermo Fisher Scientific) to a final concentration of 5 × 10^4^ cells/mL. The mixture was plated onto a 96-well μ-Plate (ibidi GmbH, Germany). Each well was cultured in either MSTC-conditioned or control (unsupplemented) medium, collected as described above in the Sample Collection section, and imaged 16 h later. The endothelial tubes were traced and quantified using Fiji and the AngioTool plugin.^13,14^

### Live/Dead viability assay

MSTCs were collected after incubation in DMEM medium for 24 h, as described above, washed with phosphate-buffered saline (PBS), then incubated in staining solution containing calcein AM (live cell label) and Propidium Iodide (dead cell label) for 30 min. The stained MSTCs were washed again in PBS, then imaged by confocal microscopy. The amount of Propidium Iodide staining in the epidermis as a function of distance from the tissue edge was quantified using Fiji^14^: 3D image stacks were compressed into 2D images by maximum intensity projection, then straight lines were traced from the edge to the center of the epidermal head of individual MSTCs, with six or more of these linear regions of interest spread evenly over the surface of each MSTC. The intensity profile was taken over each line, and to control for variations in background intensity between different tissue samples, the intensity of each pixel measured was adjusted for the local baseline intensity and normalized against the local maximum as follows:

$$I_{adj}=\frac{I-I_{min}}{I_{max}-I_{min}},$$

where *I_adj_* denotes the adjusted intensity, *I* denotes the measured intensity, whereas *I_min_* and *I_max_* denote the minimum (baseline) and maximum intensities, respectively, for the specific linear profile. In other words, the numerator (*I − I_min_*) represents the increase in intensity above baseline, whereas the denominator (*I_max_ − I_min_*) represents the dynamic range for each linear intensity profile. Adjusted linear intensity profiles are averaged for each MSTC, and reported as functions of distance from the epidermal tissue edge. To investigate how the tissue damage profile is affected by MSTC size, this analysis was repeated for MSTCs collected with 21G (∼500 μm inner diameter) and 19G (∼700 μm inner diameter) harvesting needles. Five independent MSTCs for each size were analyzed this way.

### Cytokine content analysis

To identify differences in protein release profiles between MSTC and intact full-thickness skin, intact full-thickness skin was collected from postabdominoplasty skin by excision after subcutaneous fat was removed by scraping with surgical scalpels, to simulate the typical clinical preparation of full-thickness skin grafts. Conditioned media of these samples were obtained from four independent samples, using the same mass of tissue (200 mg) and following identical collection procedures for MSTCs, as described above. Soluble proteins in conditioned media were identified and quantified using the RayBiotech (Norcross, GA) Quantibody array-based multiplex enzyme-linked immunosorbent assay (ELISA) testing service for 660 human biomarkers.

### Statistics

Statistical analysis was performed with guidance from the Harvard Catalyst Biostatistical Consulting service. GraphPad Prism version 7.0a for Mac OS X (GraphPad Software, La Jolla CA) was used for calculations. Unpaired *t*-test with Welch's correction was used to compare results from the migration, proliferation, and endothelial tube formation assays, between groups treated with conditioned media and control base media (*n* = 4). Results are reported as mean ± 95% confidence interval. *p*-Values less than 0.05 were considered statistically significant. For the comparison of soluble protein release profiles between MSTCs and intact full-thickness skin, 115 proteins with at least twofold difference between groups were compared by unpaired *t*-test with 5% false discovery rate (two-stage linear step-up method of Benjamini, Krieger, and Yekutieli).

## Results

**Figure 1.**
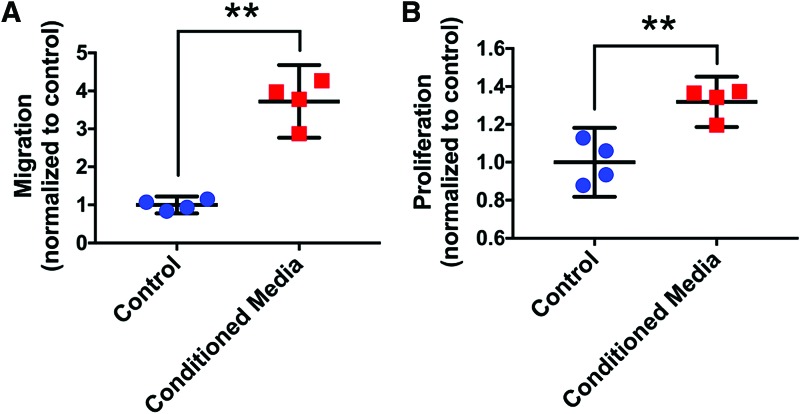
Migration **(A)** and proliferation **(B)** of HFF1 dermal fibroblasts both increased after exposure to MSTC-conditioned media for 24 h, compared with control (unsupplemented) media. Mean ± 95% CI. **p < 0.005, *n* = 4. CI, confidence interval; HFF1, human foreskin fibroblasts; MSTC, micro skin tissue column. Color images are available online.

**Figure 2.**
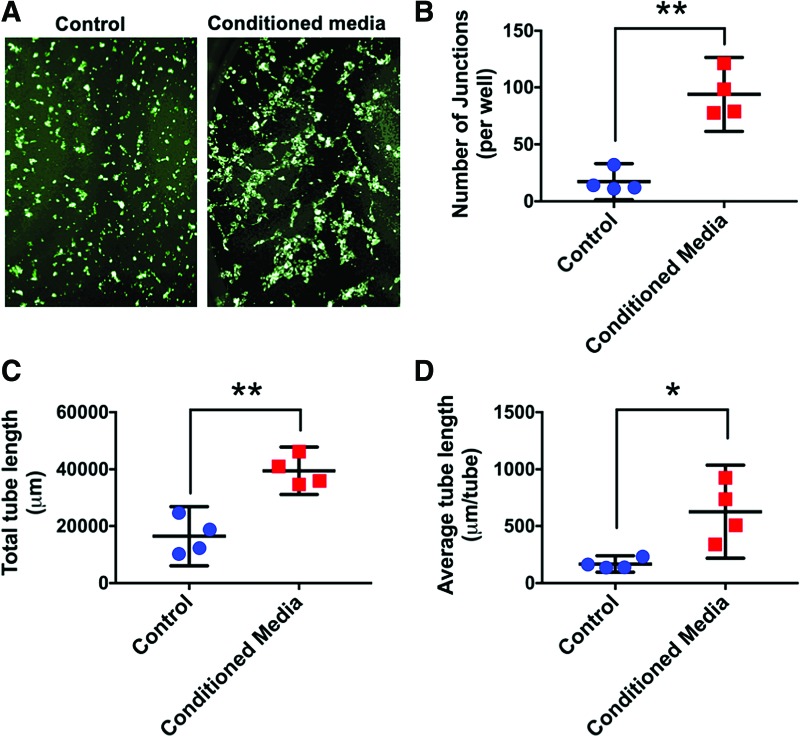
MSTC-conditioned media induced endothelial tube formation by HUVECs, but control media did not (**A**, each image width corresponds to 1.2 mm). Number of junctions **(B)**, total tube length **(C)**, and average tube length **(D)** were all significantly increased in response to the MSTC-conditioned media. Mean ± 95% CI. *p < 0.05, **p < 0.005, *n* = 4. HUVEC, human umbilical vein endothelial cell. Color images are available online.

Exposure to MSTC-conditioned media for 24 h caused significant increases in both migration and proliferation of HFF1 human dermal fibroblasts, by 3.7- and 1.3-fold, respectively, compared with unconditioned control medium (Fig. 1). MSTC-conditioned media also induced extensive tube formation by HUVEC cells, whereas no tube formation was observed following incubation with control media (Fig. 2).

**Figure 3.**
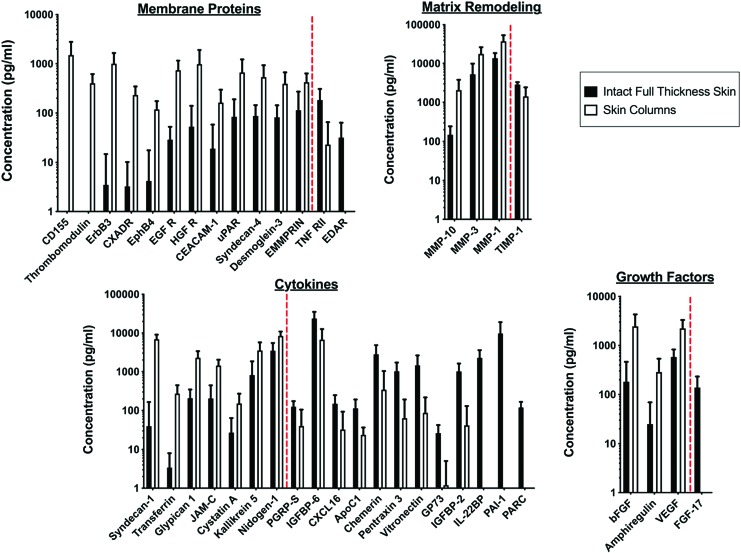
Protein release of MSTCs compared with intact full-thickness skin. Six hundred sixty proteins were measured. Twenty-five proteins were increased significantly by more than twofold in MSTC-conditioned media, whereas 16 were significantly decreased by more than twofold. These proteins are grouped into four functional categories (membrane proteins, matrix remodeling, cytokines, and growth factors), and listed on the *x*-axes of their respective graphs in order of increasing concentration ratio between intact full-thickness skin versus skin column. The *red dotted line* on each graph marks the transition between factors that were more highly released from skin columns (on the *left* of each *red line*), versus those with reduced release from skin columns (on the *right* of each *red line*), compared with intact full-thickness skin. Color images are available online.

A broad range of soluble factors were identified in MSTC-conditioned media, many of which are well known for their contributions to the wound healing process, as highlighted in Table 1. A complete list of protein release data is included in the supplement (Supplementary Table S1). Protein release profiles were generally similar between MSTCs and full-thickness intact skin (619 out of 660 proteins assayed, or ∼94%), but there were 41 proteins with over twofold difference between the two groups, including a few with substantial release from one group, but levels below detection limit in the other (Fig. 3). Among the 25 proteins with higher levels for the MSTC conditioned medium, three were growth factors known to be prominently involved in wound healing (amphiregulin, basic fibroblast growth factor, and vascular endothelial growth factor), three were matrix metalloproteinases (MMP-1, 3, and 10), and most of the remainders were membrane or intracellular proteins. In contrast, the 16 proteins with higher release levels for the intact full-thickness skin conditioned medium were mainly cytokines and a MMP inhibitor (TIMP1).

**Table 1.** Partial list of soluble proteins released by micro skin tissue columns that are known to significantly impact the wound healing process

Amphiregulin (AREG)	Matrix metalloproteinase 7 (MMP7)
Angiogenin	Matrix metalloproteinase 9 (MMP9)
Basic fibroblast growth factor (bFGF)	Matrix metalloproteinase 10 (MMP10)
Granulocyte colony-stimulating factor (G-CSF)	Midkine
Growth/differentiation factor 15 (GDF-15)	Monocyte chemoattractant protein-1 (MCP-1)
Hepatocyte growth factor (HGF)	Placental growth factor (PlGF)
Interleukin 6 (IL-6)	Tissue inhibitor of metalloproteinase 1 (TIMP-1)
Interleukin 8 (IL-8)	Tissue inhibitor of metalloproteinase 2 (TIMP-2)
Macrophage inflammatory protein 1 alpha (MIP-1α)	Transforming growth factor alpha (TGF-α)
Macrophage migration inhibitory factor (MIF)	Transforming growth factor beta-1 (TGFβ-1)
Matrix metalloproteinase 1 (MMP1)	Vascular endothelial growth factor (VEGF)
Matrix metalloproteinase 3 (MMP3)	WNT Inhibitory Factor 1 (WIF-1)

**Figure 4.**
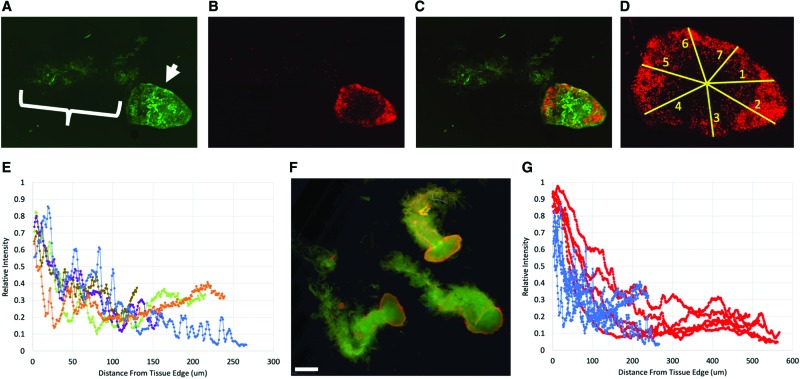
Representative image of LIVE/DEAD staining in MSTCs, after 24 h of incubation (the time point of conditioned media collection). Live cells stained with calcein AM (**A**, epidermis highlighted by *arrow*, dermis by *bracket*), dead cells with Propidium Iodide **(B)**. Superimposed image with both stains shown in **(C)**. **(D)** Example of linear region of interests (ROIs, labeled 1–7 in the image), each spanning the distance from the edge to the center of the epidermal surface of an MSTC, used to obtain Propidium Iodide staining intensity profiles. **(E)** Propidium iodide staining intensity (adjusted for baseline and normalized for dynamic range, as detailed in the text) as a function of distance from tissue edge. Each curve shows the average values from the linear ROIs for one MSTC, curves from five independent MSTCs are shown. Dead cells are predominantly located along the tissue periphery. **(F)** Live/Dead staining results in 19G MSTCs. Scale bar = 1 mm. **(G)** Propidium Iodide staining intensity as a function of distance from tissue edge, in 21G MSTCs (*blue*) and 19G MSTCs (*red*), *n* = 5 each. Overall, both sizes showed similar intensity profiles, with dead cells predominantly located along the tissue periphery. MSTC, micro skin tissue column; ROI, region of interest. Color images are available online.

Live/dead staining showed that celluar injury was most densely distributed along the tissue periphery, with the intensity of Propidium Iodide staining falling to about half its peak value within the first 50 μm from the edge of tissue (Fig. 4). This injury pattern was similar between smaller (21G) and larger (19G) MSTCs (Fig. 4F, G).

## Discussion

In this study we found that MSTCs release soluble factors that increased migration and proliferation of dermal fibroblasts, and proangiogenic factors that promote tube formation by HUVECs. These results, combined with our previous finding that a diverse range of cells and multicellular structures derived from MSTCs are engrafted stably into wound sites, show that MSTCs are able to enhance wound healing both by directly replenishing the wound volume with various autologous skin cells/structures (which is a distinctive feature of MSTCs, compared with conventional cell-based therapies, where long-term engraftment rarely occurs), and also by generating and releasing signaling molecules to influence cells from surrounding tissue to take part in the repair process.

The protein release profiles between MSTCs and full-thickness intact skin were generally similar, with the exception of a small subset of proteins which showed a different expression pattern. This latter finding is consistent with previous data showing that mincing of skin grafts causes some alterations in protein release.^15^ The increased release of membrane/intracellular proteins from MSTCs may be due to injury inflicted on cells at the edges of each MSTC during the harvesting process, which is consistent with live/dead staining in MSTCs showing cellular damage mostly distributed along the periphery (Fig. 4). The membrane proteins could also be components of extracellular vesicles, which are increasingly recognized for their ability to modulate the wound healing process.^16^ These potential sources of membrane proteins are not mutually exclusive, as apoptotic cell death triggers the release of apoptotic bodies.^17^ This and other responses to local tissue injury (*e.g.*, cell activation in the vicinity of an injury^18^) are likely to be more prominent in MSTCs or minced tissue, due to the higher total wound edge area compared with larger conventional grafts (since the wound area scales with the circumference of the tissue samples, for this study the total wound area was about 20 × lower in the 10 mm-diameter intact skin, compared with an equivalent volume of 0.5 mm-diameter MSTCs), which is likely to at least partially account for the observed differences in protein release profiles. The increased release of growth factors and MMP from MSTCs, coupled with the reduction in metallopeptidase inhibitor 1 (TIMP1), are suggestive of a shift toward a more proliferative and migratory milieu that is also consistent with a tissue injury response. Taken together, our data suggest that MSTCs may be in a more injured state compared with intact skin grafts, which carries two main implications: (1) it may be beneficial to combine MSTCs with modalities known to ameliorate cell distress associated with tissue injury and/or increase cell proliferation, for example, negative pressure wound therapy,^19^ cell salvage reagents,^20^ and some energy-based therapies,^21,22^ to increase survival of MSTC-derived cells; (2) an injured state in the donor tissue may actually be advantageous, as a previous clinical study has shown that prewounding of donor sites led to improved skin grafting outcomes,^23^ presumably due to a kick starting of tissue repair mechanisms. Further studies are needed to determine whether/how this injured state should be specifically mitigated or altered to optimize healing outcomes.

One particular challenge posed by chronic wounds is the pathologic, hostile wound environment, with abnormalities in one or more factors, including tissue oxygenation, pH, inflammatory status, microbial colonization, etc. There is accumulating evidence in the literature that wound healing can be enhanced by exposing the wounds to just the secretome from various cell-based therapies,^24^ and our results show that secreted factors from MSTCs may be able to serve a similar function. In addition, MSTCs may serve both as source and the recipient of MSTC-derived paracrine signaling. In chronic wounds, cells within the wound bed and the periwound area are often dysfunctional, as a result their ability to respond to paracrine signals may be impaired compared with normal cells. In that setting, healthy cells derived from MSTCs may be better able to respond to signaling from other MSTCs (or even internally from the same MSTC). On the other hand, initially healthy MSTCs could quickly become distressed if wound conditions prove to be overly hostile, which would most certainly impact the quality of the MSTC-derived secretome, and its ability to impart meaningful clinical effects. A better understanding of this tug-of-war between healthy MSTCs and pathologic wound conditions would be valuable for informing the optimal clinical application of this treatment approach.

Given the critical impact of cytokine signaling on the wound healing process, much effort has been devoted to developing growth factors as therapies to enhance wound healing. However, clinical outcomes from growth factor-based therapies have been disappointing, which has been attributed to various factors such as rapid degradation of applied factors in the hostile wound environment, as well as ineffective delivery mechanisms.^25^ In addition, since molecular signaling in wound healing normally occurs in a highly orchestrated and dynamic manner involving multiple different pathways, it is difficult to recapitulate using therapeutic regimens based on the static application of a single growth factor, or even a combined panel of different growth factors. Cell-based therapies have a potential advantage by synthesizing rather than merely adding signaling factors; for as long as the cells remain viable they can continuously produce, release, and regulate a multitude of signaling factors in response to local microenvironmental cues. This study shows that autologous MSTCs could fulfill a similar role by providing wounds with both cells and signaling factors to stimulate healing, but without the complex and costly production processes associated with conventional cell-based therapies.

Shortcomings of this study include *in vitro* culture conditions that by necessity cannot reproduce the complexity and dynamics of the *in vivo* wound environment, some mystery about the actual cellular source of soluble factors, as MSTCs contain the full complement of skin structures and cell types, and lack of information regarding signaling entities outside of the ones queried (other proteins, miRNAs, exosomes, etc.).

## Innovation

Autologous skin grafting is the current gold standard for wound repair, but it is limited by substantial donor-site morbidity. MSTC harvesting was recently developed as an alternative method to obtain autologous skin tissue while minimizing donor-site morbidity. The current study shows that MSTCs are able to produce various soluble factors that increase cell migration, proliferation, and vascular tube formation—thus MSTCs are able to enhance wound healing both by directly supplying autologous cells to replenish the wound volume (shown previously), and indirectly through paracrine signaling.

**Key Findings**

- MSTCs release many soluble proteins known for prominent roles in the wound healing process.
- These soluble factors significantly enhanced fibroblast migration and proliferation, and endothelial cell tube formation, in *in vitro* assays.
- The soluble factor release profile may be altered due to tissue damage caused by the harvesting procedure, in comparison to conventional full-thickness skin grafts.

## Supplementary Material

Supplemental data

## About the Authors

**Joshua Tam, PhD,** is an Instructor in Dermatology at the Wellman Center for Photomedicine. He received his doctorate in Biomedical Engineering from the Harvard-MIT Division of Health Sciences and Technology. His main research interest is in new technologies to enhance wound healing and tissue regeneration. He coinvented the technology for harvesting autologous, full-thickness skin tissue in a fractionated pattern, and using the tissue for wound repair. For this work he was awarded the 2016 Wound Healing Foundation 3M Fellowship. **Martin Purschke, PhD,** is an Instructor in Dermatology at the Wellman Center for Photomedicine. He received his doctorate in Biochemistry from the University of Oldenburg, Germany. His main research interest is the development of new technologies to improve cancer treatment. **Christiane Fuchs, PhD,** is a Research Fellow at the Wellman Center for Photomedicine. She received her doctorate in genetics and microbiology from the University of Vienna, Austria, and performed postdoctoral work on muscle tissue engineering and shockwave therapy for wound healing at the University of Applied Sciences Technikum Wien, Austria. Her current research interests include therapies to improve wound healing and mechanisms involved in skin regeneration. **Ying Wang, MD, PhD,** is a plastic surgeon with more than 10 years' experience in clinical burn and wound care. Her research focuses on wound repair using microcolumn skin grafting and autofluorescence wound imaging. She has also been involved in the infection and pain control areas. **R. Rox Anderson, MD,** is Director of the Wellman Center for Photomedicine, Professor of Dermatology at Harvard Medical School, and a fellow of the National Academy of Inventors. He invented many laser-based therapies currently in clinical use, such as lasers for vascular and pigmented lesions, permanent hair removal, tattoo removal, and scar remodeling, as well as wound treatments, including epidermal blister grafting and the full-thickness skin column grafting described in this article.

## Abbreviations and Acronyms

DMEM: Dulbecco's modified Eagle's medium
ELISA: enzyme-linked immunosorbent assay
HFF1: human foreskin fibroblasts
HUVEC: human umbilical vein endothelial cell
IRB: Institutional Review Board
MMP: matrix metalloproteinase
MSTC: micro skin tissue columns
PBS: phosphate-buffered saline
