# Supplementary material for: Skin Microcolumns as a Source of Paracrine Signaling Factors
Source: Adv Wound Care (New Rochelle). 2020 Feb 7;9(4):174–83. doi: 10.1089/wound.2019.1045 (PMC7047113; doi:10.1089/wound.2019.1045)
Supplement: Supplemental data [file Supp_Table1.pdf]

Protein	Concentration (pg/mL)							
	Full-thickness skin				Micro skin tissue columns			
2B4	0	0	12.9424037	0	0	0	0	0
4-1BB	0	0	0	0	0	0	0	0
4-1BB Ligand	0	0	0	0	0	0	0	0
6Ckine	0	0	128.8	1695.1	0	0	0	0
A2M	404.578999	281.41953	501.267905	407.619822	577.61263	977.447712	466.685619	0
ACE	0	770.948859	839.816189	1090.60236	0	1790.92547	1083.91996	502.241423
ACE-2	0	0	0	0	0	0	0	0
Activin A	0	0	0	0	0	0	0	0
Activin RIB	123.08656	0	0	0	0	0	0	0
Activin RIIB	0	0	0	0	0	0	0	0
ADAM12	0	0	0	0	0	0	0	0
ADAM23	0	0	93.0856084	181.231576	0	0	76.568982	80.0413478
ADAM8	0	0	0	0	0	0	0	0
ADAM9	0	0	5.01613476	0	0	0	0	0
ADAMTS13	0	0	1253.44587	259.913658	0	0	0	0
Adiponectin	115543.888	61520.2645	32328.824	47179.0199	147217.954	128584.107	46747.7019	34771.2978
Adipsin	9807.06934	10405.7749	7898.3326	10038.782	10213.7252	11154.6532	10140.7788	10116.2586
aFGF	0	0	0	0	0	0	0	0
AFP	0	0	0	0	0	0	0	0
Aggrecan	14.5554047	39.0785481	0	16.7421209	0	0	0	0
AgRP	0	0	0	0	0	0	0	0
Albumin	885.000741	1016.00943	11836.3101	2623.51441	2802.68903	4002.61861	4293.73767	7079.67353
ALCAM	0	0	0	0	0	7.87769747	0	0
ALK-1	64.2845639	56.0409403	244.172295	0	49.7244414	0	459.981889	0
AMICA	0	0	0	0	0	0	0	0
AMIGO	0	0	0	0	0	0	0	0
Aminopeptidase LRAP	0	0	0	0	0	0	0	0
Aminopeptidase P2	0	0	0	0	0	0	0	0
Amnionless	0	0	0	0	0	0	0	0
ANG-1	0	0	14.7264468	0	0	0	0	0
ANG-2	22.0556909	24.0917079	160.418863	49.9341501	0	63.684551	0	0
ANG-4	0	0	0	0	0	0	0	0
Angiogenin	646.75817	657.335188	828.33489	896.643067	338.649703	817.928126	1047.23401	97.2325091
Angiostatin	0	76.1192486	1600.29253	370.684231	0	0	0	0
Angiotensinogen	118.957297	124.484187	0	67.3364374	3.67287637	0	0	0
ANGPTL3	0	0	0	0	0	0	0	0
ANGPTL4	1166.11671	563.056074	5202.30634	956.806427	0	0	0	0
ApoA1	3186.57251	1182.92875	4890.29248	0	2430.96943	76.1950855	358.269367	0
ApoC1	102.388788	62.3144027	110.61825	180.911821	24.6260946	27.2717098	30.874558	12.38205
ApoC2	763.197866	0.14364046	392.304628	8.35055487	4797.35969	11.1565513	42.7045509	22.7549691
ApoC3	1.6516846	0.30827669	5.63589019	13.2865481	0.1993713	0.36280511	0.22377525	0.1628227
ApoE	122.135485	231.741175	351.800006	435.61777	138.171738	0	234.361106	66.8466307
ApoH	2067.07757	0	2586.4069	3801.23836	1970.23691	489.469949	1844.75051	1288.48643
APRIL	0	0	0	0	0	0	0	0
AR	0	64.7000899	20.6697715	14.5865445	124.600336	493.312362	219.152618	307.025494
Artemin	0	0	0	12.8752312	0	0	0	7.25481241
Arylsulfatase A	0	0	0	0	0	0	0	0
Axl	0	0	0	0	0	0	0	0
b-NGF	0	0	0	0	0	0	0	0
B2M	341.259526	219.699561	273.560076	227.188995	330.680627	203.932874	464.672331	316.542934
B7-1	0	0	0	0	0	30.9975341	0	0
B7-2	13.4828406	13.0252878	1.94236351	0	6.26943216	0	0	8.76269246
B7-H1	0	0	0	0	0	0	0	0
B7-H2	0	2.8222934	0	0	0.32579877	0	0	0
B7-H3	0	0	8.16808318	100.979571	0	0	4.72081004	15.7769827
BAFF	0	0	0	0	0	0	0	0

**BAFF R**	0	0	0	0	0	0	0	0
**BAMBI**	0	3.34441193	0	14.1465772	0	0	0	0
**BCAM**	70.704515	203.112598	303.41467	71.4021367	383.344931	641.1336	1506.09824	1056.38238
**Bcl-w**	0	0	0	0	0	0	0	0
**BCMA**	9.03477792	50.9587452	55.6981113	82.2902306	0	37.5760383	0	0
**BDNF**	0	0	0	1.41333438	0	0	0	0
**bFGF**	217.96214	410.957296	0	96.3199153	1281.2293	3779.08995	1727.38728	3048.80625
**bIG-H3**	7334.81723	7244.69381	2737.60986	3437.05425	7811.09701	8755.02746	4396.83896	3288.8589
**BLAME**	106.06399	94.101702	0	22.9957038	78.103997	0	0	0
**BLC**	0	0.03750761	0	0	0	0	0	0
**BMP-2**	6.21599909	0	0	0	0	0	0	0
**BMP-4**	3.68086998	0	0	0	0	0	0	0
**BMP-5**	0	0	0	0	0	0	0	0
**BMP-7**	0	0	0	0	0	0	0	0
**BMP-8**	62.5360728	167.078227	0	0	0	0	0	0
**BMP-9**	0	0	0	0	0	0	0	0
**BMPR-IA**	0	77.3037664	74.0870816	0	0	0	0	0
**BMPR-IB**	0	0	0	0	0	0	0	0
**BMPR-II**	0	0	312.999975	93.8687259	0	0	0	55.2565177
**BOC**	0	0	0	0	0	0	0	0
**Brevican**	0	0	15.3201677	18.93406	6.19525258	103.693247	225.092873	0
**BTC**	0	0	0	0	0	0	0	0
**C5a**	61.7353605	405.07011	638.356984	511.136201	51.0577491	12.5662254	56.4788226	0
**CA125**	0	0	0	0	264.019605	0	0	0
**CA15-3**	0	0	0	0	0	0	0	0
**CA19-9**	0	0	0	0	0	0	0	0
**CA9**	520.33101	561.924443	256.790484	280.243463	1128.66084	1371.51056	791.039987	497.736711
**Cadherin-11**	0	0	0	0	0	0	0	0
**Cadherin-13**	0	0	0	0	0	0	0	0
**Cadherin-4**	0	0	0	0	0	0	0	0
**Calcitonin**	0	0	0	0	0	0	0	0
**Calsyntenin-1**	105.815392	28.4928522	0	0	0	0	0	0
**Carbonic Anhydrase XII**	0	0	0	5034.12146	0	0	0	0
**Carboxypeptidase A2**	0	0	0	0	0	0	0	0
**Cardiotrophin-1**	0	0	284.986538	781.432137	0	0	0	0
**Cathepsin B**	104.326002	7.0120933	105.721619	194.214937	9.78392059	0	125.45651	0
**Cathepsin E**	3.17115994	3.02538652	0	0	0	0	0	0
**Cathepsin L**	1104.70958	707.134349	2553.23837	1404.06951	404.135989	925.987917	1165.34147	69.2887125
**Cathepsin S**	383.689069	601.548123	127.167143	155.139554	60.872106	584.571989	0	0
**Cathepsin V**	0	0	0	0	0	0	0	0
**CCL28**	0	0	0	0	0	0	0	0
**CD109**	0	0	2409.90705	2872.38433	0	0	0	0
**CD14**	1841.1842	2283.98879	4415.71592	4907.44557	1154.0357	2067.53966	2578.40802	1950.91104
**CD155**	0	0	0	0	1514.09954	1953.24286	2213.39718	366.891889
**CD157**	0	4.50881017	47.2069387	0	0	0	0	0
**CD163**	2514.32731	16173.0266	8153.85116	19490.2446	3222.65735	16550.9862	874.446019	1299.90043
**CD200**	215.122932	0	0	0	0	0	0	0
**CD229**	6.08173109	0	0	0	0	0	0	0
**CD23**	0	22.8994561	0	0	0	0	0	0
**CD27**	16.5967965	17.9994993	1.61364217	0.27690186	0	0	0	0
**CD28**	0	0	0	280.252191	0	0	0	0
**CD30**	0	0	0	0	0	0	0	0
**CD300c**	0	28.9528689	0	154.953715	0	0	0	0
**CD320**	96.2343006	0	0	1252.08011	0	0	0	0
**CD34**	0	0	0	0	0	0	0	0
**CD40**	0	0	0	0	0	0	0	0
**CD40L**	1.9860922	2.61520241	0	6.21632342	0	0	0	0
**CD48**	0	0	0	0	0	0	0	0

**CD58**	0	207.579738	0	84.3701797	422.061327	601.368061	19.5784475	0
**CD6**	0	0	0	0	0	0	0	0
**CD83**	0	0	0	0	0	0	0	0
**CD84**	0	0	0	0	0	0	0	0
**CD97**	0	324.19324	0	232.934808	0	0	0	0
**CD99**	376.912352	429.094863	425.025934	538.74099	507.207196	577.79205	550.548332	508.772114
**CDNF**	0	0	4.47674973	16.6895462	0	0	0	0
**CDO**	10.8432553	98.379832	136.678898	414.974294	0	164.950613	118.709519	15.3577215
**CEA**	0	0	0	0	0	0	0	0
**CEACAM-1**	24.2391136	0	0	52.0184127	192.262333	266.964268	113.103384	77.8480033
**CEACAM-5**	0	0	0	0	0	0	0	0
**CF VII**	0	44.3640028	0	0	0	0	0	0
**CF XIV**	122.914117	0	0	0	0	0	0	0
**Chemerin**	4018.41182	3751.72631	1304.56846	2156.06776	16.6444876	921.366829	442.180139	0
**CHI3L1**	937.527631	980.255412	414.140569	328.429317	993.413069	1685.875	927.897595	732.703397
**CHST1**	63.1863668	252.306855	44.9656016	626.798414	0	0	35.4363101	0
**CHST4**	0	312.306229	33.0970236	1010.05135	0	0	0	0
**cIAP-2**	0	84.2889497	61.464065	0	0	0	0	0
**CILP-1**	0	125.939551	431.650778	958.995605	0	0	32.5378448	19.1004929
**Ck beta 8-1**	14.4952121	15.8622711	0	36.2272626	0	0	0	0
**CLEC-1**	0	0	0	0	0	0	0	0
**CLEC10A**	0	0	0	0	0	0	0	0
**Clusterin**	69.9532457	73.5770304	74.0331692	4.92742665	0	126.796818	0	0
**cMASP3**	157.91687	144.228061	0	0	93.6010358	0	0	0
**CMG-2**	0	0	0	0	0	0	0	0
**CNTF**	0	0	0	0	0	0	0	0
**CNTF R alpha**	0	0	0	0	0	0	0	0
**Common beta Chain**	0	0	53.9769275	11.8885995	0	0	8.29206091	0
**Contactin-1**	36.525459	46.987697	17.9102638	34.2785598	39.4318935	42.1843726	68.2719076	42.1059398
**CREG**	0	0	0	0	0	0	0	0
**CRIM1**	0	1.68772993	19.9572637	16.7815999	262.85752	159.464099	99.8483177	31.6468967
**Cripto-1**	10.6578293	13.5842619	11.0056553	13.8363551	0	0	0	0
**CRP**	71.0590972	310.956909	120.368638	2311.9764	8.23950282	113.064114	9.10780177	365.748038
**CRTAC1**	0	0	0	1364.33798	0	0	0	0
**CRTAM**	0	0	0	0	0	0	0	0
**CTACK**	91.5882457	250.62692	969.1	531.9	0	19.5895703	39.9	11.7
**CTLA4**	0	0	0	0	0	0	0	0
**CXADR**	0	3.9333831	0	9.22145095	233.311893	258.354165	302.425393	135.619108
**CXCL14**	0	0	0	0	0	0	0	0
**CXCL16**	73.4644966	155.5611	226.7	141.9	0	73.676364	56.6	0
**Cystatin A**	5.16049659	27.9799296	58.8602611	16.1708658	241.282593	184.316698	110.206179	70.595333
**Cystatin B**	2112.25809	2491.77113	1161.63653	1751.33664	3274.26139	2909.3045	1654.68778	1406.34608
**Cystatin C**	643.124486	1201.98697	768.411382	1033.90889	326.582898	745.635869	439.297076	204.243013
**Cystatin E M**	504.567617	430.747683	568.69715	672.094963	1115.13327	896.596046	1030.8718	1006.37202
**Cystatin SN**	0	0	0	0	0	0	0	0
**Cytokeratin-8**	0	0	0	0	0	0	0	0
**DAN**	0	0	0	0	0	0	0	0
**DcR3**	0	0	0	0	0	0	0	0
**Decorin**	2672.76901	3169.1585	2876.13652	3428.14169	3065.06821	2818.26778	3113.39959	3075.8258
**Dectin-1**	0	0	0	0	0	0	0	0
**Desmocollin-3**	0	0	0	0	0	0	0	0
**Desmoglein 2**	0	0	0	0	0	0	0	0
**Desmoglein-1**	47.7930028	52.97418	92.7755663	30.9727041	143.126913	70.3265808	307.23432	94.2754896
**Desmoglein-3**	111.218231	116.795519	37.3668241	61.7001054	436.671868	600.497537	347.360446	182.695085
**DKK-1**	0	0	0	0	0	0	0	0
**Dkk-3**	533.064282	1002.25457	756.236713	558.064434	2025.6876	974.116244	714.160003	199.363897
**Dkk-4**	0	0.90890409	0	0	0	0	0	0
**DLL1**	0	0	0	0	0	559.243543	0	0

**DNAM-1**	0	0	0	0	0	0	0	0
**Dopa Decarboxylase**	0	9.38131246	10.9243424	3.9874832	0	0	0	0
**DPPII**	307.939001	2012.37739	513.507123	1079.50718	4792.82923	10976.5226	2757.92027	3033.13483
**DPPIV**	2349.03507	3060.96926	2120.0118	2603.076	11492.2779	13988.198	4256.54045	2254.87165
**DR3**	0	0	0	0	0	0	0	0
**DR6**	0	0	0.68289157	1.47209535	0	0	1.84170732	47.3456234
**DSPG3**	0	20.4806949	0	0	0	0	0	0
**Dtk**	0	24.2884918	0	0	0	0	0	0
**E-Cadherin**	0	0	0	0	0	0	0	0
**E-Selectin**	603.841102	0	0	2.65291633	457.00619	398.040613	0	139.021441
**ECM-1**	0.71714647	1.06942291	23.2620971	44.4363414	0	0.10136266	0.08342515	2.45440649
**EDA-A2**	0	0	0	0	0	0	0	0
**EDAR**	45.9997977	51.4086272	10.080224	19.5926523	0	0	0	0
**EG-VEGF**	0	0	0	24.5617308	0	0	9.40647947	1.66334833
**EGF**	0	0	0.35471644	0	0	0	0.0061784	0.00395623
**EGF R**	9.00170887	34.5189753	44.1578287	28.0998112	562.339961	807.522214	1084.80414	505.794328
**EMMPRIN**	28.3958479	43.5731667	242.735347	140.806642	350.160729	309.012927	613.104321	409.411289
**EMR2**	0	2.83572104	17.4070673	3.55172942	0	0	0	0
**ENA-78**	2133.12582	1465.91774	581.7	153.5	65.3742333	1436.40968	122	0
**Endocan**	0	0	0	0	3.31619018	0.62933178	0	0.42436842
**Endoglin**	0	0	0	0	0	8.9555163	0.19584342	4.35029356
**Endoglycan**	0	0	0	0	0	0	0	0
**Eotaxin**	2.82319592	1.00792313	0	0	0	0	0	0
**Eotaxin-2**	0	0	0	0	0	0	0	0
**Eotaxin-3**	0	0	0	0	0	0	0	0
**EpCAM**	0	0	0	0	0	0	0	0
**EphA1**	321.598747	167.807855	0	29.9173287	344.843363	0	61.360191	0
**EphA2**	17.6574324	0	37.4171481	0	66.5761594	42.8102892	51.4110658	29.7460734
**EphB4**	0	0	0	16.8784001	124.969321	77.1554744	161.753301	109.673529
**EphB6**	89.2680685	39.1132207	0	40.7224078	0	0	0	0
**Ephrin-A4**	146.094048	87.1585171	0	0	133.577742	0	0	0
**Ephrin-B3**	493.129336	537.891077	929.448185	2494.84231	612.925095	578.792158	1252.0801	959.290023
**Epiregulin**	860.652722	1281.4501	0	0	369.99666	96.5521689	0	0
**Epo R**	0	40.5631941	0	0	0	0	0	0
**ErbB2**	0	0	5.2381835	0	0	0	0	0
**ErbB3**	14.1008698	0	0	0	1365.74849	947.289431	1251.00634	443.688333
**ErbB4**	0	0	67.8529532	0	0	0	10.0549373	0
**ESAM**	0	0	0	0	0	0	0	0
**FABP1**	0	0	91.2158379	0	0	0	0	0
**FABP2**	12.4625459	36.3260463	0	63.4919775	0	0	0	0
**FAP**	72.2490562	78.8391636	207.092002	182.696435	124.638948	303.345148	370.41528	83.0111325
**Fas**	9.19774136	0	0	0	4.11885237	9.46315811	0	1.4360483
**FAS L**	0	0	0	0	0	0	0	0
**FCAR**	0	0	0	10.2998924	0	0	0	0
**Fcg RIIBC**	78.7632701	169.783743	0	165.163442	0	0	0	0
**FCRL1**	0	0	0	64.5480604	0	0	0	0
**FCRL2**	0	0	0	0	0	0	0	0
**Ferritin**	0	0	84168.4973	68763.124	0	0	88757.3564	25112.1677
**Fetuin A**	47548.1715	51043.6243	65656.7881	33867.9364	31948.461	38220.0073	27071.4196	7237.2992
**FGF-12**	29.8868355	29.5207157	0	0	31.6605152	5.71104347	0	0
**FGF-17**	185.003757	192.880866	94.3452561	80.125259	0	0	0	0
**FGF-19**	0	0	0	0	0	0	0	0
**FGF-20**	0	0	0	7.71369775	0	0	1.12824903	9.58863876
**FGF-21**	0	0	0	0	0	0	0	0
**FGF-23**	54.095293	0	3.8626458	0	0	0	0	0
**FGF-4**	0	0	0	0	0	0	72.6720579	0
**FGF-5**	0	0	0	0	0	0	0	0
**FGF-6**	2.58777094	58.4033555	0	0	0	0	0	0

**FGF-7**	0	0	0	0	0	0	0	0
**FGF-9**	9.49318126	13.5402633	0	0	0	0	0	0
**FLRG**	124.24098	59.4236066	389.978772	80.5031691	50.0827157	79.8611717	30.5611845	0
**Flt-3**	0	13.265661	60.3640144	0	0	0	0	0
**Flt-3L**	0.89595619	0.67002636	5.5059111	1.86561462	0.45089882	7.2744469	0	1.28311912
**Follistatin**	31.9863758	178.834756	24.9063433	81.9110777	0	0	0	0
**Follistatin-like 1**	0	3331.35556	0	0	0	0	0	0
**FOLR1**	0	0	0	0	0	0	0	0
**FOLR2**	189.478186	1440.06258	516.570843	533.116432	678.025849	1631.3171	407.771696	303.163951
**Fractalkine**	22.1797427	4.30720087	0	0	0	0	0	0
**FSH**	0	0	0	0	0	0	0	0
**Furin**	0	0	501.747135	0	0	0	0	0
**G-CSF**	156.067725	115.794636	431.519049	151.252454	97.5280103	610.806222	124.584469	21.3272492
**G-CSF R**	0	0	0	0	0	0	0	0
**Galectin-1**	156.902111	245.400411	2312.47557	2712.0274	212.432928	331.97394	2220.96836	2071.37311
**Galectin-2**	0	0	0	0	0	0	0	0
**Galectin-3**	3166.81446	3392.76484	3161.51089	3934.98176	1779.22464	3861.40003	907.940774	1937.41515
**Galectin-4**	0	0	38.3854004	0	0	0	0	0
**Galectin-7**	58155.6534	87407.8274	13337.805	15422.6349	89651.1813	109040.287	13543.318	11409.9964
**Galectin-8**	75.1776412	33.6320811	1.51108522	0	200.544229	311.960907	33.8178991	16.3751012
**Galectin-9**	252.764128	1484.16013	598.452491	1057.48485	1445.42273	2525.5429	1681.27098	1547.44223
**Gas 1**	0	3.60705007	0	0	0	0	0	0
**Gas6**	0	5.56083153	0	0	0	0	0	0
**GASP-1**	0	0	0	0	0	0	0	0
**GASP-2**	0	0	0	0	0	0	0	0
**GCP-2**	45.8703653	50.2545336	0	0	0	0	0	0
**GDF-15**	31.4153698	31.9399452	47.4671946	96.8530391	45.2181876	80.9198333	72.966982	52.8338888
**GDF-8**	0	0	240.921789	164.816054	0	0	0	0
**GDNF**	0	0	16.138878	0	0	0	14.8898047	0
**GH**	0	0	0	0	0	0	2.90390789	0
**GHR**	46.6517954	75.5155663	27.5689555	224.541223	13.0343293	29.5342188	7.50528868	0.85083914
**GITR**	0	0	0	0	0	0	0	0
**GITR L**	0	0	0	0	0	0	0	0
**GLP-1**	0	0	0	0	0	0	0	0
**Glypican 1**	108.818114	284.163411	280.548334	162.33178	1902.18517	2857.30659	2868.5273	1507.82951
**Glypican 2**	0	0	0	0	0	0	0	0
**Glypican 5**	291.95056	370.239599	0	268.902541	88.8684869	219.982077	0	0
**GM-CSF**	5.0972957	1.02568971	0	0	0.52636972	0	0.24514293	0.05628223
**GM-CSF Ra**	0	0	0	0	0	0	0	0
**gp130**	121.097242	0	75.8738575	0	0	0	0	0
**GP73**	34.4523374	28.5221702	11.7912737	30.8370376	0	0	0	4.75997542
**GPR56**	0	8.20515369	0	70.707712	0	0	0	0
**GPVI**	0	12.4422156	23.329665	31.2651785	0	52.1757907	50.8778964	15.8122133
**Granulysin**	0	0	0	0	0	0	0	0
**GRO**	6.5624422	5.97450393	262.3	329.7	3.32455202	4.21698064	184.5	91.5
**GROa**	83102.6424	84701.2063	39565.8199	54999.3537	14727.7725	96114.4454	25842.6116	4214.81402
**HAI-1**	0	0	357.577388	786.092384	0	0	352.654309	0
**HAI-2**	0	0	0	0	0	0	0	0
**HAPLN1**	0	0	0	0	0	0	0	0
**HB-EGF**	0	0	0	0	0	0	0	0
**HCC-1**	663.340082	694.985355	213.5	200.2	45.7099049	264.539221	66	14.4
**HCC-4**	0	0	0	0	0	0	0	0
**hCGb**	0	0	0	0	0	0	0	0
**Hepsin**	0	46.7294859	0	0	0	0	0	0
**HGF**	41.2008829	11.5831518	142.819777	199.123767	69.680526	96.367032	178.141256	221.704734
**HGF R**	41.1916489	0	129.296464	40.8496263	1034.17591	1672.54525	977.843826	251.353507
**HTRA2**	204.61434	227.798348	1020.00272	675.063753	271.318634	697.261634	511.986996	112.669518
**HVEM**	0	0	0	0	0	0	0	3.29964611

**I-309**	7.33710216	0	0	0	0	0	0	0
**I-TAC**	0	0	23.6	0	0	0	0	0
**ICAM-1**	155.809791	282.802185	231.575716	299.162403	398.181603	803.231281	472.647826	305.474693
**ICAM-2**	227.773414	197.485392	982.393052	332.285855	0	0	0	0
**ICAM-3**	0	0	0	0	0	0	0	0
**ICOS**	0	0	0	0	0	0	0	0
**IFN-gamma R1**	43.1417211	13.9518248	0	4.86223774	0	0	0	0
**IFNab R2**	0	0	0	0	0	0	0	0
**IFNg**	1.17801281	0.63516384	0	0	0	0	5.56235854	0.93035413
**IgA**	14804.5045	23988.1041	41730.6078	66098.3883	20022.9399	37269.5091	12400.7225	13564.5323
**IgD**	2416.16947	2721.28329	462.03848	793.667108	4949.40268	2165.63442	954.523091	30.0767011
**IgE**	2107.78112	236.268813	834.402425	720.4493	3567.25707	2272.96155	771.973748	1325.13707
**IGF-1**	0	0	0	0	0	0	0	0
**IGF-1R**	0	0	0	0	0	0	0	0
**IGF-2**	24.9351033	98.5183254	0	0	0	0	0	0
**IGF-2R**	0	0	0	0	0	0	0	0
**IGFBP-1**	0	116.081968	21.6206402	0	0	99.9024006	8.07125951	0
**IGFBP-2**	1440.56733	1164.24905	911.788811	552.182669	0	48.9020459	0	117.337519
**IGFBP-3**	662.329714	111.111832	836.857769	879.736513	0	0	59.7919948	874.975286
**IGFBP-4**	664.907657	1983.52549	0	0	3241.88107	5100.86966	0	0
**IGFBP-5**	598.37105	0	0	0	0	0	0	0
**IGFBP-6**	18442.8601	20730.5432	21360.7313	34640.8759	1769.04598	8460.7594	10573.4325	6288.08284
**IgG1**	19925.9064	70904.9022	35391.1243	56810.4538	55593.8974	99085.8558	29834.3371	6948.08169
**IgG2**	4144.18293	26804.5109	0	0	28620.9887	32784.9844	4344.03198	2857.28627
**IgG3**	638.810008	1076.78737	374.865616	187.332607	939.328403	1365.8538	516.356884	31.7899934
**IgG4**	3438.85343	2370.56698	376.459553	1011.32163	2183.50039	4872.17974	113.992398	195.135266
**IgM**	3485.9746	7118.15863	4940.95226	4156.31267	6189.68542	10260.8582	2416.3047	1084.3097
**IL-1 F10**	0	0	0	0	0	0	0	0
**IL-1 F5**	0	0	0	0	171.989833	51.5206411	1.43352742	33.4588349
**IL-1 F6**	467.135553	449.029821	0	0	0	0	0	0
**IL-1 F7**	256.182057	612.893222	255.736738	0	39.8538511	362.367794	255.150324	248.316773
**IL-1 F8**	0	0	0	0	0	0	0	0
**IL-1 F9**	1762.82662	646.051538	0	0	1954.05906	1898.72553	0	0
**IL-1 R3**	89.5637609	108.905875	240.850835	493.207666	52.2946781	71.4699476	57.2467581	8.55765735
**IL-1 R4**	0	8.4953395	148.315221	0	571.001562	2214.08694	4551.3537	859.664805
**IL-1 R5**	0	0	0	0	0	0	0	0
**IL-1 R6**	0	0	0	0	0	0	0	0
**IL-1 RI**	3.1834847	0	0	0	0.06300473	1.95027	0	0
**IL-1 RII**	0	0	0	0	463.445307	243.529944	906.02522	0.98031864
**IL-10**	1.09807472	0.62646096	0	0	0	2.17384459	0.23340425	0
**IL-10 Ra**	0	0	0	0	0	0	0	0
**IL-10 Rb**	0	0	0	0	0	0	0	0
**IL-11**	10.9052768	12.3665821	0	0	0	0	0	0
**IL-12p40**	0.56103018	0.58601894	2.12324928	0	0	0	0	0.07453738
**IL-12p70**	0.29006599	0.3506308	0	0	0	0	0	0
**IL-13**	18.9431607	12.3268318	0.2190329	0	0	1.20343573	1.09349412	0.30529413
**IL-13 R1**	0	0	0	0	0	0	0	0
**IL-13 R2**	0	0	0	0	0	0	0	0
**IL-15**	12.6120676	5.60913933	0	0	0	0	15.2698423	0
**IL-15 R**	0	0	0	0	0	0	0	0
**IL-16**	14.8681514	22.8214942	13.720995	8.8490245	17.0326163	35.7031114	13.1516346	0
**IL-17**	6.37954321	0	0	0	0	0	0	0
**IL-17B**	0	0	7.34295393	0	0	0	0	0
**IL-17B R**	0	0	0	0	0	0	0	0
**IL-17C**	0	0	0	0	0	0	0	0
**IL-17E**	18.8590301	0	0	0	0	0	0	0
**IL-17F**	0	0	0	0	0	2.06941101	0	0
**IL-17R**	0	0	0	7.02429549	0	0	0	0.84153296

**IL-18**	0	0	0	24.5444363	15.8171347	0	1.40598986	20.7870937
**IL-18 BPa**	0	0	0	0	0	0	0	0
**IL-18 Rb**	0	0	0	0	0	0	0	0
**IL-1a**	6.37345585	5.30687961	0	0	14.5290611	24.4815387	0	0
**IL-1b**	18.0485827	19.3065728	52.1898352	52.9584695	13.5716183	14.9011325	63.2931918	49.5119432
**IL-1ra**	41.8972977	72.3771984	0	0	160.82707	245.430558	28.5218755	10.2449192
**IL-2**	1.53085788	1.2880128	0	0	0	0	2.30719881	0
**IL-2 Ra**	0	0	0	0	0	0	0	0
**IL-2 Rb**	0	0	0	0	0	0	0	0
**IL-2 Rg**	225.492212	0	914.105072	426.826173	371.374862	626.50003	1041.48365	598.934117
**IL-20**	0	13.8557238	0	0	2.04318233	67.1657887	0	0
**IL-20 Ra**	0	0	0	0	0	0	0	0
**IL-21**	0	0	0	0	0	0	0	0
**IL-21R**	0	0	0	0	0	44.8340693	0	0
**IL-22 R alpha 1**	65.4944809	41.9495969	11.3755547	44.3060281	36.5123628	48.1850232	6.49323658	0
**IL-22BP**	1559.28519	3217.71237	2697.57633	1606.5841	0	0	0	0
**IL-23**	0	0	39.9945851	28.8231445	0	0	0	0
**IL-23 R**	21.1584063	31.7610607	21.4971898	31.9641442	18.7279762	19.7788286	24.0263889	0
**IL-24**	0	0	0	0	0	77.645148	0	0
**IL-27**	0	0	0	0	0	0	0	0
**IL-27 Ra**	0	0	79.5099172	0	0	0	0	0
**IL-28A**	0	0.08480148	0	0	0	1.40642734	0	0
**IL-29**	0	0	0	0	0	0	0	0
**IL-3**	0	0	8.93183162	4.24262685	0	0	0	0
**IL-31**	143.560918	40.8329406	0	0	0	0	0	0
**IL-31 RA**	80.7627116	29.7643935	16.0276434	38.0972723	22.0067787	0	0	0
**IL-32 alpha**	0	0	0	0	0	0	0	0
**IL-33**	0	27.1112311	114.947386	147.508764	0.06823498	333.662872	24.7368175	42.9147371
**IL-34**	0	0	0	0	0	0	0	0
**IL-4**	2.74230113	0	0	0	0	0	0	0
**IL-4 Ra**	9.86190329	14.63259	2.3267981	0	12.890898	25.302187	11.0059385	5.02184453
**IL-5**	36.4591835	25.4321639	0	0	0	0	0	0
**IL-5 Ra**	0	0	0	0	0	0	0	0
**IL-6**	682.925779	837.364339	179.903286	141.612516	558.250548	882.203786	154.956009	97.6646583
**IL-6R**	49.7547976	51.2583899	70.1116313	133.881104	40.5824047	60.0969755	90.3378099	37.1288999
**IL-7**	6.31545963	3.02530326	0	0	0	0	0.59313794	0
**IL-7 R alpha**	28.2563468	46.0848146	41.0152814	105.807696	18.0100186	32.7464988	9.66722252	4.0364337
**IL-8**	138.162717	111.739574	37.2204049	16.6175097	228.881436	333.879881	16.1993447	42.6446641
**IL-9**	179.513541	136.53164	0	0	0	0	0	0
**ILT2**	0	0	9.9428453	19.4334104	0	0	0	0
**Insulin**	0	0	0	0	0	0	0	0
**Insulin R**	0	0	89.5903177	230.305204	0	0	59.8399921	0
**Integrin alpha 5**	0	0	0	0	0	0	0	0
**IP-10**	2.18929456	0	0	0	0	0	2.7	0
**Jagged 1**	0	0	0	0	0	0	0	0
**Jagged 2**	0	0	0	171.810924	0	0	0	0
**JAM-A**	0	0.24688906	0	19.9955458	0	15.6985931	17.9621426	0
**JAM-B**	0	0	0	0	0	0	0	0
**JAM-C**	52.622955	216.638285	408.294217	144.355705	977.418479	1887.33925	1421.17075	1485.61993
**Kallikrein 14**	23.7071858	4.90789059	15.0867573	4.08636479	0	4.62157937	0	0
**Kallikrein 5**	447.477493	634.350289	1773.17729	419.078854	4118.16044	3746.57125	4741.90956	1490.3887
**Kallikrein 7**	148.815429	242.91979	43.4168522	106.92599	722.166262	650.117845	484.514412	180.195705
**Kirrel3**	0	0	0	145.419116	0	0	0	0
**KLF4**	0	0	0	710.77618	0	0	0	0
**L-Selectin**	0	0	296.320416	651.847575	0	0	0	0
**L1CAM-2**	0	0	0	0	0	0	0	0
**LAG-3**	0	0	0	0	0	0	0	0
**LAIR1**	0	0	0	173.359063	0	0	0	0

**LAMP**	0	129.706504	0	0	0	0	0	0
**LAMP1**	0	20.7476972	0	307.023177	0	751.519288	641.112027	353.331919
**Langerin**	0	0	0	0	0	0	0	0
**LAP(TGFb1)**	27.6921147	42.8075854	82.233267	43.3521686	134.504979	163.020095	88.6291538	0
**Layilin**	0	0	0	0	0	0	0	0
**LDL R**	0	0	0	0	59.5703007	84.7056412	26.2853715	3.72588054
**Legumain**	4221.02911	2196.51325	3835.57295	4116.56401	8083.17547	9008.89119	6828.71294	2176.95154
**Leptin**	457.129929	22.410836	127.530592	29.7420523	0	26.3841823	0	0
**Leptin R**	0	0	0	0	0	0	0	0
**LH**	95.8574329	32.3360874	0	0	0	0	0	0
**LIF**	16.9455693	0	0	0	0	0	0	0
**LIF R alpha**	0	0	23.8225703	28.2556927	0	0	0	0
**LIGHT**	0	10.8181314	0	0	0	0	0	0
**LIMPII**	0	0	0.1225736	0	0	12.6408164	69.9409852	16.0273718
**Lipocalin-1**	8.49759669	14.3634043	0.31415208	0.04971683	0	0	0	0
**Lipocalin-2**	52.1397896	367.957712	221.290951	184.917735	525.980339	1144.33852	697.54971	154.840769
**LOX-1**	0	0	0	0	0	0	0	0
**LRIG3**	0	100.714874	0	0	0	0	0	0
**LRP-6**	0	307.694934	22.177533	0	0	0	0	0
**LTbR**	0	0	4.71657498	2.18829373	0	0	8.26249242	0.30996094
**Lumican**	1883.02917	3395.39601	4531.72307	2939.82481	1021.81706	2806.28427	1104.86444	3040.02285
**Lymphotactin**	54.7928578	0	0	0	0	0	0	0
**LYVE-1**	328.783186	280.170885	484.78425	492.407353	305.179244	288.447019	396.307895	293.982828
**Marapsin**	0	0	0	0	96.5339872	91.4696622	130.296827	0
**Matrilin-3**	0	0	0	0	0	0	0	0
**Matriptase**	0	0	0	0	0	0	0	0
**MBL**	207.141375	89.2604223	130.902998	224.380502	231.936602	97.1561421	81.1065881	34.7835112
**MCP-1**	343.751597	338.519959	164.881384	203.084552	247.491864	353.911135	162.881918	62.6990215
**MCP-2**	1.42916728	4.68448006	0	0	0	0	0	0
**MCP-3**	0	1.79515154	0	0	0	0	0	0
**MCP-4**	1.75146453	0	0	0	0	0.04115385	0	0
**MCSF**	1.32626831	0.7990134	0	0.97794543	0	0	0	0
**MCSF R**	203.595609	410.42288	590.16903	1527.82703	135.380721	349.198195	272.726815	76.9343041
**MDC**	29.0671292	116.529452	202.6	76.8	0	10.1399157	20.3	0
**MDGA1**	0	0	0	149.057271	0	0	0	0
**MDM2**	77.7673281	129.879452	0	0	0	0	0	0
**MEP1B**	0	0	0	0	0	0	0	0
**MEPE**	0	0	0	0	0	0	0	0
**MeprinA**	580.822347	60.0682557	0	0	167.26844	0	0	0
**Mer**	0	0	0	0	0	0	0	0
**Mesothelin**	0	132.092014	115.662224	41.1027621	0	42.8796902	0	0
**MFRP**	0	0	0	0	0	0	0	0
**MICA**	8.18211362	7.07364716	38.6351516	33.7600275	6.95814336	10.0503072	44.2726556	43.9236255
**MICB**	0	39.8699825	0	0	0	0	0	0
**Midkine**	36.6498345	97.4798478	65.4532313	79.5471639	31.4280491	35.7929643	40.9484294	24.5904838
**MIF**	68.8188811	136.24068	9004.5	3151.2	112.041996	333.032845	2679.5	1144.8
**MIG**	554.226237	731.258038	14.0788742	64.0383203	110.587118	202.475558	13.3669939	11.6122442
**MIP-1a**	138.878176	25.318684	33.8054922	83.7287627	0	18.4647791	2.55609474	3.40037716
**MIP-1b**	26.2973931	7.20931834	1.57201723	12.2237052	2.18981716	2.67193898	0	0
**MIP-1d**	5.40409476	6.40059276	0	0	0	0	0	0
**MIP-3a**	1.03040094	0	3.6	0	0	0	0	0
**MIP-3b**	2.21219672	0	0	0	0	0	0	0
**MIS RII**	0	0	0	0	0	0	0	0
**MMP-1**	13665.2565	17675.5355	13093.8863	10536.866	41540.9474	44396.6035	42122.1088	22827.1264
**MMP-10**	108.444107	107.417983	142.370904	234.46238	2722.80837	3010.5318	1981.94434	574.047069
**MMP-13**	0	0	159.040796	126.765163	0	0	83.4269203	0
**MMP-2**	0	0	0	0	3326.36961	0	0	0
**MMP-3**	9559.59089	3849.67629	3562.18101	4096.55815	22264.4491	20598.3068	17192.392	10144.6662

**MMP-7**	844.481743	680.184246	1509.93702	340.385731	413.314226	398.030773	515.616143	0
**MMP-8**	0	0	0	0	0	0	0	0
**MMP-9**	285.717555	306.105239	589.035952	2380.72327	693.28512	2624.13746	1236.58705	167.131717
**MPIF-1**	0	0	0	0	0	0	0	0
**MSP**	88.6173793	0	50.4	8.3	0	0	0	0
**MSP R**	0	60.1919444	0	0	19.5510591	0	0	11.460007
**N-Cadherin**	0	364.239936	18.6068632	0	24.2197423	0	0	0
**NAP-2**	29.9383883	23.9387303	286.3	40.4	7.0373982	20.6908487	13.7	2.2
**NCAM-1**	0	0	75.8347803	0	0	0	0	0
**Nectin-1**	807.785783	789.687336	0	1259.14008	0	0	0	0
**Nectin-3**	0	0	0	0	0	0	0	0
**Nectin-4**	0	0	0	0	0	0	0	0
**Neprilysin**	0	0	0	0	0	0	0	0
**Neprilysin-2**	381.67501	613.98897	0	0	388.085206	0	0	0
**Neurexin 3 beta**	0	19.5261992	0	0	0	0	0	0
**Neuropilin-2**	0	0	54.0072736	0	0	0	0	0
**Neurturin**	0	0	24.7910393	23.7222328	0	0	0	7.86895786
**NGF R**	23.4673831	0	0	1.76224957	0	0	0	6.47242459
**Nidogen-1**	3604.00735	1611.55258	4700.16829	3912.5672	6422.48615	9821.48804	9642.64084	7847.25454
**Nidogen-2**	0	0	376.710987	62.7194455	0	0	706.517576	355.691024
**NKp30**	15.9921179	110.112804	1.51507032	0	0	0	0	0
**NKp44**	0	0	0	0	8.14958995	0	0	0
**Nogo Receptor**	464.103383	410.083794	0	76.7116617	0	0	0	0
**Notch-1**	0	0	0	0	0	0	0	0
**Notch-3**	0	0	23.0153335	6.39675603	57.1785667	54.5104651	47.5824038	3.3042726
**NOV**	330.241644	404.243372	653.864998	236.720578	308.035511	908.884177	375.36226	98.2982153
**NrCAM**	48.1009446	0	0	0	0	0	0	0
**NRG1-b1**	0	0	0	2.48406465	0	0	0	0.2572272
**NSE**	1991.75922	4215.74463	3070.13829	2044.29247	8062.06748	8254.32034	2719.30581	1599.4383
**NT-3**	0	14.0534643	0	0	0	0	0	0
**NT-4**	0	0	4.04600079	5.3565005	0	0	3.07888547	7.06856448
**Olfactomedin-2**	0	0	217.228324	116.57513	0	0	0	0
**OPG**	24.6241809	2.02155335	2.20808255	13.503906	14.8390253	55.0942	16.0101594	11.8451485
**OPN**	3991.29872	8.23295998	0	79.5	0	0	0	0
**OSM**	3.30019471	26.5931616	0	0	0	0	0	0
**OSM R beta**	0	947.252662	0	320.119109	0	0	0	0
**Osteoactivin**	51.5473223	274.620828	276.704911	180.421201	109.42653	436.8922	234.822745	106.781472
**OX40**	0	0	0	0	0	0	0	0
**OX40 Ligand**	0	0	0	0	0	0	0	0
**P-Cadherin**	661.607687	540.820817	347.128807	240.933493	10428.7401	15316.8208	3711.16135	1283.80919
**p27**	0	0	0	0	0	0	0	0
**p53**	0	0	26.6809456	16.152717	0	0	0	16.6394919
**PAI-1**	4581.43651	4923.46256	13277.6712	16326.7199	0	0	0	0
**PAPP-A**	0	0	0	0	0	0	0	0
**Pappalysin-2**	0	0	0	0	0	0	0	0
**PARC**	105.465092	93.2890436	161.6	120.8	0	0	0	0
**PD-1**	0	0	0	0	0	0	0	0
**PD-ECGF**	0	424.554052	262.432488	396.571793	120.530289	308.630994	125.081196	127.973178
**PDGF Rb**	0	0	0	0	0	0	0	0
**PDGF-AA**	1.5576205	0	0.73691634	0.99817449	0	0.2811844	11.245506	3.53044229
**PDGF-AB**	0	0	0	0	0	0	0	0
**PDGF-BB**	0	0	0	0	0	0	0	0
**PDGF-CC**	618.130762	378.73212	21.9579002	38.45734	0	51.0547352	0	0
**PECAM-1**	0	0	0	0	0	0	0	0
**Pentraxin 3**	1484.03986	1208.3399	956.153986	467.73782	172.710768	77.7959571	4.93886114	0
**Pepsinogen I**	0	0	0	0	0	0	0	0
**Pepsinogen II**	18.0298213	3.40334744	0	0	0	0	0	0
**Periostin**	0	0	310.273061	40.0172598	47.8084158	72.9270398	14.5466823	0.71846769

**Persephin**	0	0	0	0	0	0	0	0
**PF4**	41.3491282	51.1211281	7847.4	123.3	0	0	65.4	0
**PGRP-S**	94.434474	138.514719	105.867165	163.263594	78.5292814	72.909617	8.20154927	1.18027789
**PIGF**	13.8434722	6.33477286	7.78978557	1.62018998	4.9617621	13.9721623	20.6341012	9.10254816
**Plexin B3**	0	0	13.9027902	0	0	0	0	0
**Plexin D1**	0	0	0	0	0	0	0	0
**Pref-1**	0	0	0	0	0	0	0	0
**Presenilin 1**	0	0	0	0	0	0	0	0
**Procalcitonin**	0	0	0	0	0	0	0	0
**Progranulin**	1470.10722	1158.99844	1568.27672	1254.99554	8220.70733	4747.42231	5846.1854	827.935275
**proGRP**	0	0	0	0	0	0	0	0
**Prolactin**	0	0	0	0	0	0	0	0
**Prolactin R**	58.1549811	0	0	29.9936282	0	0	0	0
**Prostasin**	0	0	0	0	0	0	11.5480512	0.6032745
**PSA-free**	0	0	0	0	0	0	0	0
**PSA-total**	0	0	0	0	0	0	0	0
**PSMA**	0	0	0	0	0	0	0	0
**PTH**	30.2543384	31.5918442	0	0	0	0	0	0
**PYY**	0	0	0	0	0	0	0	0
**RAGE**	0	0	0	0	0	0	0	0
**RANK**	0	0	0	0	0	0	0	0
**RANTES**	10.6330388	12.1802527	146.432058	42.9436897	7.46414338	13.2857095	39.9143207	43.1139188
**RBP4**	5988.65225	6175.36058	3116.95171	3339.27813	6492.09462	6040.65745	3292.97495	2873.40684
**Reg1B**	0	0	0	0	0	0	0	0
**RELT**	91.3047782	86.8152341	13.8768277	17.1558381	66.4213251	122.965638	38.8105765	0.28467032
**Renin**	0	0	0	0	0	0	0	0
**Resistin**	0	0	0	0	0	0	0	0
**Ret**	0	0	122.476442	719.925575	0	0	176.279533	0
**RGM-A**	0	0	0	0	0	0	0	0
**RGM-B**	0	0	68.5288552	0	0	0	0	0
**ROBO2**	0	0	0	0	0	0	0	0
**ROBO3**	4.78886124	6.37964003	13.5657082	4.80778558	0	0	0	0
**ROBO4**	0	0	0	43.0522494	0	0	123.817959	35.4074181
**Ryk**	68.9472453	106.613168	0	29.4530947	0	16.4243188	0	0
**S100A8**	0	4.83082673	0	0	0	0	0	0
**SAA**	60.2105746	0	105.496628	23.3835746	0	0	16.0882832	0
**SCF**	2.35355749	0	0.69808111	0	0	0	0	0
**SCF R**	75.0032791	65.9634768	188.182564	369.540151	188.749298	180.814694	249.653196	232.110936
**SDF-1a**	0	0	0	0	0	0	0	0
**SDF-1b**	0	0	0	0	0	0	0	0
**Semaphorin 6B**	5.66333621	0	0	0.9731036	0	0	0	8.32410898
**Semaphorin 6D**	0	585.957657	0	266.972517	0	49.0203298	0	0
**Semaphorin 7A**	133.942913	178.616687	0	0	0	28.8120514	0	0
**Serpin A4**	6007.49982	7064.59782	7635.68782	8871.87191	6519.56858	7090.25541	9257.81473	6110.67868
**Serpin F1**	0	0	0	85.1621655	0	0	124.238676	0
**sFRP-3**	178.088065	0	194.780948	0	0	71.7320826	0	0
**Shh-N**	0	0	0	0	0	0	0	0
**SIGIRR**	0	0	0	0	0	0	0	0
**Siglec-10**	0	337.178516	0	963.421283	793.285408	903.94806	148.642921	308.11643
**Siglec-11**	64.0086268	1949.21274	0	0	129.162682	143.443997	0	0
**Siglec-5**	941.636108	6928.46738	1147.54534	464.429053	0	2065.61461	0	0
**Siglec-7**	0	0	0	0	0	0	0	0
**Siglec-9**	0	0	0	0	0	0	0	0
**SLAM**	0	0	0	0	0	0	0	0
**SOST**	0	0	0	0	0	0	0	0
**SOX2**	0	6.28711882	249.569886	25.5125591	0	4.19600601	0	0
**SP-D**	0	0	0	0	0	0	0	0
**Spinesin**	0	0	0	0	0	0	0	0

**SREC-I**	16.8581578	35.8358123	78.5901247	17.9555013	16.9080689	83.3645407	61.5200195	0
**SREC-II**	0	521.907252	558.343726	954.554402	257.525011	1808.21911	4021.60562	1935.87308
**ST2**	0	0	62.5317623	0	0	2452.81503	2078.14378	0
**Syndecan-1**	0	0	158.830861	0	7935.19496	5280.38445	8145.40876	6561.80072
**Syndecan-3**	0	0	962.705991	0	0	0	0	0
**Syndecan-4**	105.498609	118.65772	87.7986851	37.0986721	723.264096	769.478033	382.031312	270.06132
**TACE**	0	0	276.065568	0	0	0	0	0
**TACI**	0	0	0	0	0	0	0	0
**TARC**	0	2.39170967	0	0	0	0	0	0
**TECK**	16.2935358	0	0	0	0	0	0	0
**Testican 2**	0	0	0	0	0	0	0	0
**TF**	0	0.29660715	0	0	0.82650926	0	0	0
**TFF3**	223.240995	101.20684	1480.71914	72.2273444	0	0	0	0
**TFPI**	0	0	0	0	0	0	0	0
**TFPI-2**	245.171652	259.030872	57.8493463	0	18.9116992	137.82353	97.8270429	3.02329443
**TfR**	47.8102011	28.1581458	96.5078863	98.7176189	4.53570387	11.1795254	37.783105	21.3627001
**TGFa**	0	0	0.05897761	0.01992996	0.38515095	54.3391724	13.2545268	8.35515118
**TGFb RIII**	0	0	0	0	0	0	0	0
**TGFb1**	0	0	0	204.725866	0	0	71.4248485	380.269101
**TGFb2**	0	0	0	0	0	0	0	0
**TGFb3**	0	0	0	0	0	0	0	0
**Thrombomodulin**	0	0	0	0	399.019391	439.020407	546.856636	223.358502
**Thrombospondin-2**	657.37901	1199.28788	2283.4095	3224.3698	1924.00687	2948.03278	3259.20974	1090.75588
**Thrombospondin-5**	9.07955026	3.93672628	0	0	0	0	0	0
**Thyroglobulin**	0	0	0	0	0	0	0	0
**Tie-1**	0	1.05506295	0	0	0	0	0	0
**Tie-2**	0	0	0	0	0	0	0	0
**TIM-1**	0	0	0	0	0	0	0	16.9796884
**TIM-3**	0	0	0	32.5037622	0	0	0	0
**TIMP-1**	3018.79525	3169.58422	2573.41926	2736.50839	1594.78298	2040.81693	1511.71464	514.494657
**TIMP-2**	11064.0944	12485.6525	15453.4877	19570.049	5800.1861	9082.95936	10472.3731	6369.68812
**TIMP-4**	0	0	13.9957749	0	0	0	0	0
**TLR1**	0	0	0	11.272163	0	1.00371938	13.4583572	2.61731769
**TLR2**	8.51650505	0	0	0	0	0	0	0
**TLR3**	0	0	0	24.359697	0	6.21574099	27.2144291	13.4405828
**TLR4**	0	0	0	0	0	0	0	0
**TNF RI**	431.375847	443.440847	1548.23673	1429.13393	231.039686	192.072698	1542.6103	1140.31449
**TNF RII**	160.2943	272.976936	88.9790918	212.916073	0	0	48.5675935	43.3910174
**TNFa**	96.8126007	42.0407103	0	1.18385817	7.05960151	3.83000481	32.7203596	15.0210995
**TNFb**	72.2401254	52.6776921	0	0	16.424547	15.8378516	27.579412	20.6760564
**TPO**	9.76944973	0	0	84.8573329	0	0	0	0
**TPP1**	0	0	335.872546	491.736043	735.268805	336.026691	733.073041	172.335746
**TRACP**	331.815551	562.430606	0	0	0	0	0	0
**TRAIL**	0	0	0	0	0	0	0	0
**TRAIL R1**	0	0	0	0	0	0	0	0
**TRAIL R2**	0	0	0	0	0	0	0	0
**TRAIL R3**	0	0	0	0	0	0	0	0
**TRAIL R4**	0	0	0	0	0	0	0	0
**TRANCE**	0	0	0	0	0	0	0	0
**Transferrin**	4.95451027	6.45801077	2.21357262	0	320.642772	370.140518	283.986275	116.479945
**Trappin-2**	4667.09204	4034.20726	6175.78814	4722.11025	4910.76445	5624.992	6610.75297	5951.23138
**TREM-1**	0	0	0	0	0	0	0	0
**TREM-2**	0	0	10.5645605	8.54464466	0	0	20.4201388	0
**TrkC**	0	0	18.6705417	0	0	0	0	0
**Troponin I**	0	0	0	0	0	0	0	0
**TROY**	0	0	0	0	0	0	0	0
**TSH**	0	0	0	0	0	0	0	0
**TSLP**	2.24251844	2.98684033	0	0	0	0	0	0

**TSP-1**	11354.9532	9478.71161	24169.158	7850.49676	8295.4935	19809.9776	16925.4904	5415.8719
**TWEAK**	72.1869647	63.2900213	0	63.9762825	231.106491	300.426449	64.4072909	0
**TWEAK R**	0	0	0	0	0	0	0	0
**Ubiquitin+1**	0	0	0	0	0	0	0	0
**ULBP-1**	0	0	0	0	0	0	0	0
**ULBP-2**	0	0.58665099	0	0	0	0	0	0
**ULBP-3**	0	0	0	0	0	0	0	0
**uPA**	0	0	0	60.9799911	310.966052	5941.41057	7124.5805	2279.44266
**uPAR**	38.9262421	66.2433558	48.7286503	182.113788	564.410285	1171.80194	590.270321	337.81097
**Uromodulin**	0	0	146.44821	98.0299566	0	0	28.0634577	24.9703045
**VCAM-1**	0	0	283.078474	0	0	0	60.7366627	0
**VE-Cadherin**	0	0	0	0	0	0	0	0
**VEGF**	348.483609	678.363895	645.296414	646.413224	1883.35418	3091.46936	2435.11688	1539.38342
**VEGF R1**	204.049977	0	545.667957	230.34863	0	0	0	0
**VEGF R2**	0	0	12.9615232	5.06240316	0	0	0	8.65376235
**VEGF R3**	0	0	0	0	0	0	0	3.47892519
**VEGF-C**	0	0	0	0	0	0	0	0
**VEGF-D**	0	0	0	0	0	0	0	0
**Vitronectin**	2248.05174	982.375575	713.666491	1887.04865	206.770138	76.2869182	41.3260457	26.3839976
**vWF**	0	37.6773712	929.292933	0	192.577338	276.434598	118.467768	0
**WIF-1**	150.471782	156.092501	686.461016	211.324495	0	126.091829	92.3919129	0
**WISP-1**	0	0	0	0	0	0	0	0
**XEDAR**	0	12.017968	0	0	0	12.6356114	0	0
**XIAP**	0	0	0	0	0	659.647774	0	0
